# Verification of dose calculations with a clinical treatment planning system based on a point kernel dose engine

**DOI:** 10.1120/jacmp.v3i2.2579

**Published:** 2002-03-01

**Authors:** Lars Weber, Per Nilsson

**Affiliations:** ^1^ MDS Nordion AB Box 1704, SE‐751 47 Uppsala Sweden; ^2^ Lund University Hospital Radiation Physics SE‐22185 Lund Sweden

**Keywords:** dosimetry, point kernel, collapsed cone, treatment planning

## Abstract

Dose calculations with a collapsed cone algorithm implemented in a clinical treatment planning system have been studied. The algorithm has been evaluated in homogeneous as well as in heterogeneous media, and the results have been compared to measurements and Monte Carlo simulations. Commonly encountered clinical beam configurations as well as more complex geometries have been pursued to test the limitations of the model. The results show that the accuracy level reached allows for clinical use. Some situations, e.g., large wedge beams and dose calculations in the build up region, not specific to the collapsed cone model, show deviations (outside ±3%) compared to measurements.

PACS number(s): 87.53.Bn, 87.53.–j, 87.66.–a

## I. INTRODUCTION

Modern treatment planning techniques use highly advanced tools in order to conform the dose distribution to the target, e.g., irregular beams shaped by multileaf collimators (MLC), asymmetric beams, and noncoplanar beams. The requirements for achieving accurate dose calculations have resulted in treatment planning systems (TPS) utilizing calculations based on convolution techniques.^1^ ^–^ ^4^ To achieve high accuracy, any algorithm needs to take into account several different dose components, e.g., primary dose, dose from charged particle contamination, head scatter dose, and phantom scatter dose. In order to achieve simplicity and speed in the dose calculations, models based on pencil‐beam kernels are frequently used for dose calculations. These kernels are generated by integrating Monte Carlo (MC) calculated point kernels (energy deposition in an infinite medium around a primary photon interaction point) over depth. A number of previous investigations^5^ ^–^ ^11^ studied the accuracy and limitations under various conditions with this particular type of model^00012^ implemented in a commercial TPS (Helax‐TMS, MDS Nordion Therapy Systems, Uppsala, Sweden).

The results are satisfactory for most clinical treatment situations. However, there are still cases where the results from the pencil‐beam algorithm may be outside accepted accuracy levels (see Discussion). The inability of the model to correctly handle changes in scatter from lateral heterogeneities, together with the lack of scaling of the electron transport, can result in significant dosimetric errors in, e.g., the thoracic region.^7^ The integration volume for phantom‐scatter calculation is dependent on phantom/patient contour and can, in some cases, give rise to deviations between measured and calculated doses, as has been shown in specific phantom geometries.^6^ ^,^ ^8^ Another problem is related to the spatially invariant pencil‐beam kernel not handling off‐axis softening.^5^ Incorrect dose calculations in the build up region5 have also been noted.

A different approach with the potential to solve a number of specific pencil kernel deficits is the point kernel method for energy deposition calculations. The collapsed cone method originally developed by Ahnesjö^13^ has been implemented into a TPS.^14^ ^,^ ^15^

**Table I:** Summary of characterization data

Type of measurement	Purpose
Central axis depth doses in water for four field sizes.	Energy spectrum derivation; modeling of charged particle contamination; verification calculations
Dose profiles in X and Y direction at five depths in water for the same four field sizes as above.	Source size modeling; verification calculations
Star shape scanned dose profiles at isocenter depth in water	Energy fluence and wedge modulation distribution
Output factors in water	Energy spectrum derivation; verification calculations
Output factors in air	Head scatter modeling; monitor back scatter derivation
Absolute dose measurement	TPS dose calibration

The collapsed cone method is a specific approximation to the point kernel approach. The kernels are discretized in a number of directions, unevenly distributed in angle with a concentration in the forward direction where most of the photons are scattered.^16^ For each direction, the kernel h is analytically described by an exponential over *r* ^2^, *r* being the distance from the point of interaction, i.e.,

$$h(r)=\frac{Ae^{-ar}+Be^{-br}}{r^{2}},$$

where *A, a, B*, and b are fitting parameters depending on the scattering angle. The dose deposited during the ray trace is distributed into the patient volume according to the specifications in the point kernels. All energy emitted in a solid angle cone is assumed to be transported along the cone axis; hence the name collapsed cone.

The aim of the present work is a general dosimetric evaluation of the collapsed cone algorithm implemented in the Helax‐TMS treatment planning system. Furthermore, a number of situations where the pencil‐beam kernel algorithm has shown deficiencies are created to test the limitations of the model. Results from the comparisons for two photon energies, and the accuracy limits associated with the model, will be presented.

## II. METHODS AND MATERIALS

### A. Test parameters

As the implemented collapsed cone model is an alternative method to the pencil‐beam model to deposit energy in the irradiated medium, the focus has been in this area. No particular focus has been given to issues related to how the TPS models head scatter or charged particle contamination. A number of measurements have been made to assess the implementation of the collapsed cone algorithm in the TPS and these include (i) output in water and in air; (ii) depth doses; and (iii) profiles.

In addition, a set of calculations is included that focuses on geometries where the pencil‐beam model has shortcomings. These measurements also include investigations to test the model limitations regarding (iv) off‐axis softening; (v) partial phantom irradiation; and (vi) heterogeneities.

The basic treatment unit characterization is the same as for the pencil‐beam model implemented in the TPS, and a summary of the measurements are listed in Table I.

The data in Table I serve as input for a process that converts the characteristics of the beam quality in terms of parameters used during subsequent dose calculations in the TPS for the actual treatment beam configuration.

### B. Measurements

The measurements were performed on an Elekta SL^i^ plus (Elekta, Crawley, UK) accelerator having photon energies of 6 and 18 MV The accelerator has a source‐to‐axis difference (SAD) of 100 cm, a distance at which all output factor measurements were carried out. The measurements in water were all performed at an source‐to‐surface distance (SSD) of 90 cm.

Depth doses and profiles at 10–cm depth were measured with a standard radiation field analyzer. Depth dose scans were made using a small volume $\left(120{\text{mm}}^{3}\right)$ ionization chamber (Scanditronix Medical, Uppsala, Sweden). For the profile measurements, a photon diode (Scanditronix Medical, Uppsala, Sweden) was used. The radiation field analyzer (Scanditronix Medical, Uppsala, Sweden) was also used as a positioning device for output measurements at off‐axis locations.

Output factors at isocenter in water were obtained using a Baldwin‐Farmer (BF) type ionization chamber $\left(600{\text{mm}}^{3}\right)$ (NE Technology, Beenham, UK) connected to an electrometer (MDS Nordion, Uppsala, Sweden). For the smallest field sizes where the large volume of the BF chamber would compromise the measurements, the small volume ionization chamber was used instead. This chamber was also used for measurements at off‐axis positions. Output factors at isocenter in air were measured using the BF chamber provided with a high density build up cap of brass having a wall thickness of 2.5 mm for the lower energy and a lead cap with 6‐mm wall thickness for the higher energy.^17^

### C. Treatment planning system calculations

Calculations were performed with Helax‐TMS version 5.1 on a 667 MHz Compaq XP1000 workstation (Compaq, Houston, TX). A standard 10 10 × 10 cm field calculated on a 30 cmX30 cm X 30 cm phantom with 61 slices takes 6.75 min, including database writing of the results. The calculation time is virtually independent of dose grid resolution (see the Appendix for a more detailed discussion of dose calculation time properties). The calculation times are longer compared to pencil‐beam calculations where the same beam arrangement takes only 45 s with the same dose grid. The calculation time for the collapsed cone algorithm is proportional to the number of voxels times the number of cone directions in the kernel tessellation. The point kernel used during this investigation has a total number of 106 cones, with 6 cones in the backward direction, 40 sideways, and 60 in the forward direction.

In order to achieve the best resolution for the line dose calculations, phantoms of different sizes were used. A margin of 5 cm was always maintained around the field edges to assure full phantom scatter at the point of calculation.

## III. RESULTS

All results are presented as output factor normalized dose, $d_{i}^{\text{OFN}}$, if not otherwise stated:

$$d_{i}^{\text{OFN}}=\frac{{\left(D(r)/M\right)}_{i}}{{\left(D/M\right)}_{\text{calib}}},$$

where *D*(**r**)/*M* is the dose at position r per monitor unit for a given beam *i*. ${\left(D/M\right)}_{\text{calib}}$ is the dose per monitor unit for the calibration field for that beam quality. Note that this normalization retains all absolute dose properties and avoids any ambiguities related to normalization. The calibration geometry used is a 10 cm×10 cm field, SSD=100 cm at depths 5 cm for 6 MV and 10 cm for 18 MV.

Deviations are presented as local percentage deviations:

$$\Delta d=100\cdot \frac{{\left(D/M\right)}_{\text{calc}}-{\left(D/M\right)}_{\text{meas}}}{{\left(D/M\right)}_{\text{meas}}}[\%].$$

As the data sets for 6 and 18 MV show similar results, only the 6‐MV data set will be presented. However, where it is of particular interest the 18 MV data will be shown. A complete data set may be requested from the corresponding author, including graphs for both energies.

### A. Output factors

Output factors in water and in air have been measured for a range of field sizes, symmetrical as well as asymmetrical fields. A total number of 30 open and 26 wedge field output factors have been acquired. Output factors for fields having an offset are always measured and calculated at the geometrical center of the beam.

**Figure 1:**
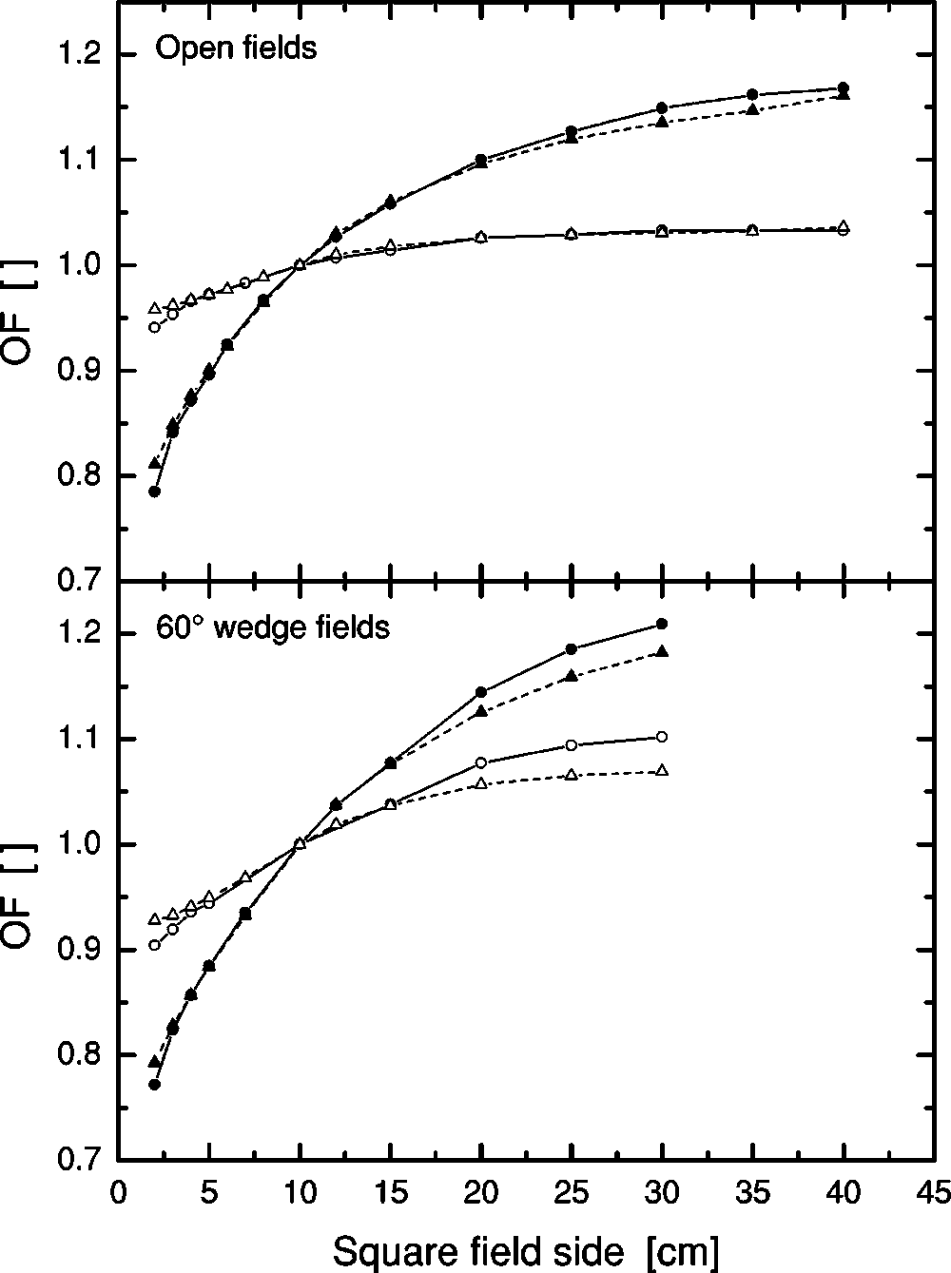
6‐MV output factors in water and in air for open (upper panel) and 60° wedge (lower panel) square fields normalized to 10 10 × 10 cm. The lines indicate measurements (solid, —) and calculations (dashed, ‐ ‐ ‐). Symbols indicate total dose (filled, ●,▲) and head scatter dose (open, ○,Δ).

**Figure 2:**
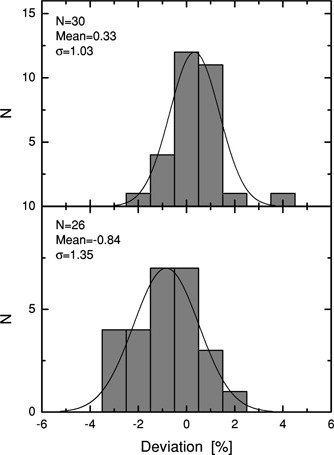
Deviations for output factors in water at 6 MV for a range of different field configurations ranging from standard square fields to highly asymmetric fields. Upper panel, open fields; lower panel, 60° wedge fields.

Figure 1 shows output factors in water and in air for square fields, normalized to a 10 10 × 10 cm field having the same modulation type. An output factor normalized summary for a range of different field configurations ranging from standard square fields to highly asymmetric fields is shown in Fig. 2. For open fields, the agreement is within or close to 3%. Only the smallest fields have larger deviations.

The wedged field calculations have larger deviations as compared to open beam calculations (Fig. 2).

### B. Depth doses

**Figure 3:**
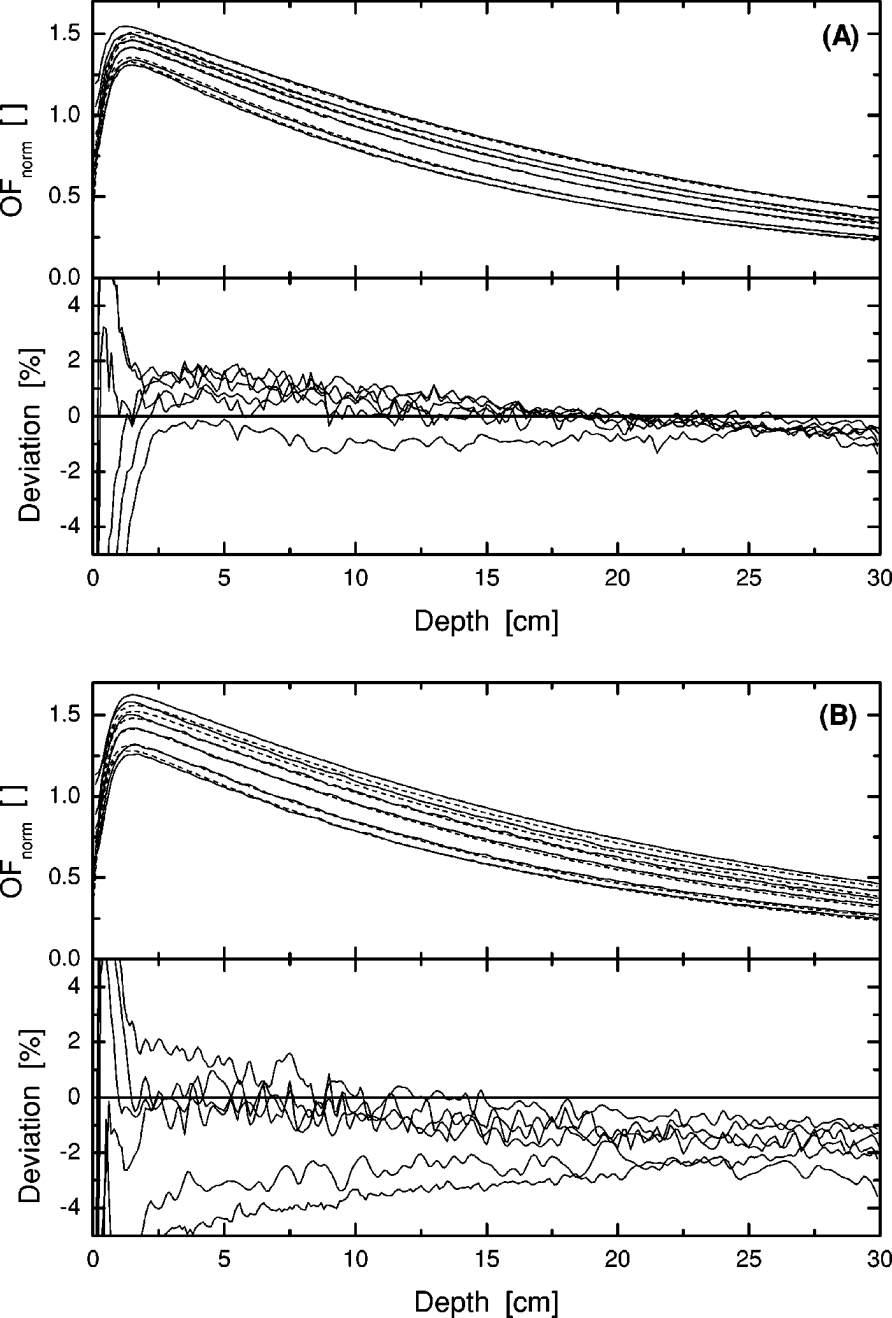
Depth doses for 6 MV open (A) and 60° wedge square fields (B). The deviation between calculated and measured data is shown in the lower part of each panel. The lines indicate measurements (solid —) and calculations (dashed ‐ ‐ ‐) at SSD=90 cm. (A) Square open fields with field sides 35, 20, 15, 10, 5, and 3 cm (top to bottom). (B) Square 60° wedge fields with field sides 30, 20, 15, 10, 5, and 3 cm (top to bottom).

Figure 3(a) shows depth doses for open beams and these are all reconstructed well compared to measurements. With the wedge applied, Fig. 3(b), the dose for the two largest fields, are underestimated as already seen in the output data, Fig. 1. If, however, the data is presented with a normalization at 5‐ and 10‐cm depth for 6 and 18 MV, respectively, the agreement is enhanced considerably. The maximum difference between calculations and measurements for the 6 MV, 60° wedge fields would then amount to 1.6% over the field size range presented in Fig. 3(b), indicating that the major source of error is related to the output factor for the largest wedged fields.

### C. Profiles

**Figure 4:**
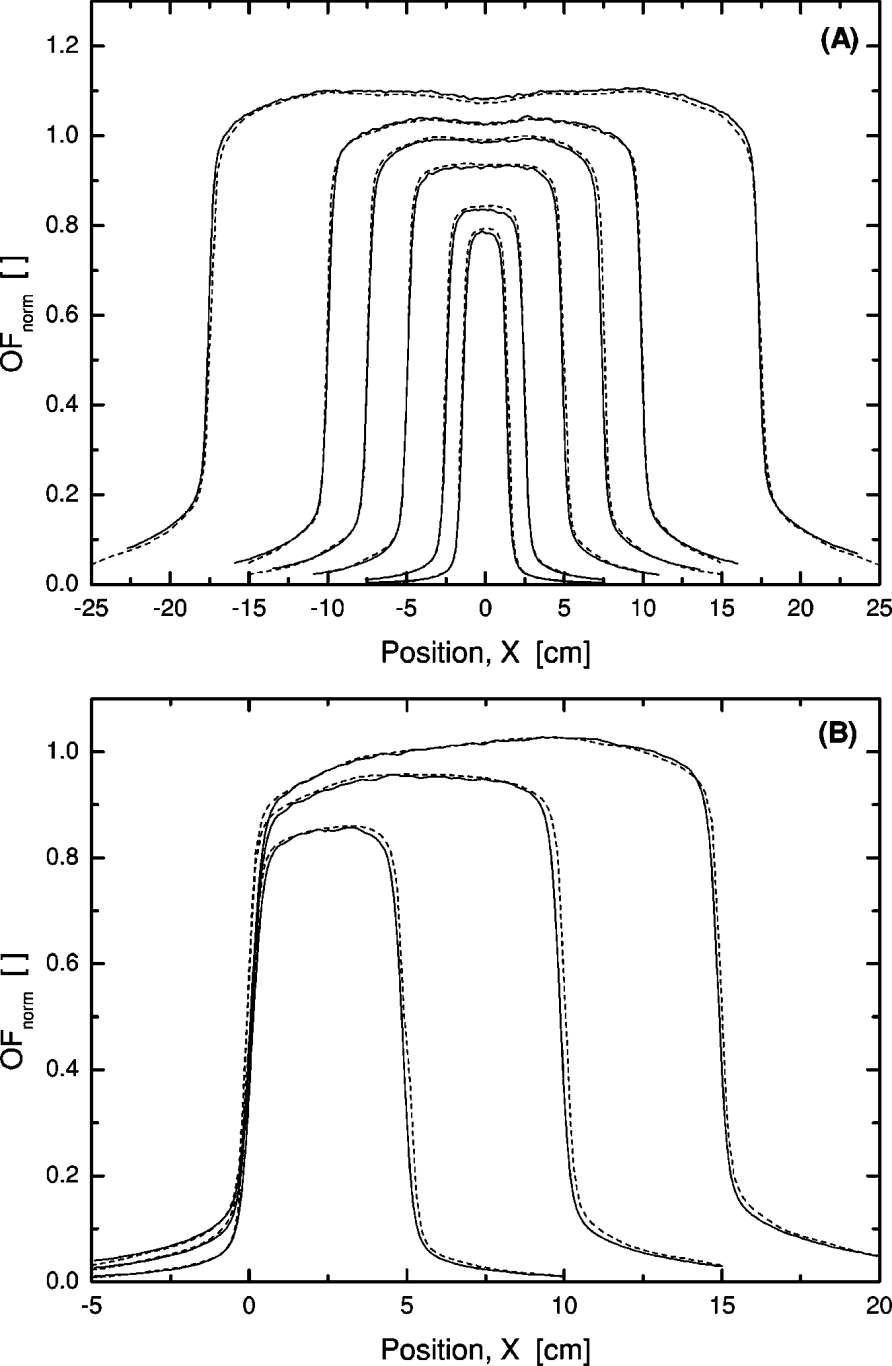
Profiles for open fields at 10 cm depth, 6 MV, SSD=90 cm. (A) Square fields with field sides 35, 20, 15, 10, 5, and 3 cm (top to bottom). (B) Square fields with field sides 5, 10, and 15 cm with offset (2.5,0), (5,0), and (7.5,0) cm, respectively. The lines indicate measurements (solid –) and calculations (dashed ‐‐‐).

Data for symmetric open beams are presented for square fields in Fig. 4(a) whereas beams with an offset and having a common jaw position at the center (X=0) are shown in Fig. 4(b). The agreement with measurements is good except locally close to the field edges. This can be explained by the fact that the accelerator is equipped with an MLC and the jaws and leafs may be slightly misaligned. Although the leaf and jaw calibration complies with manufacturer specifications and IEC^19^ standards, the settings differ slightly as compared to TPS calculated values where the nominal value is assumed. The jaw settings are within 1.5 mm of the nominal field border, whereas the TPS calculated field border is within 0.5 mm.

**Figure 5:**
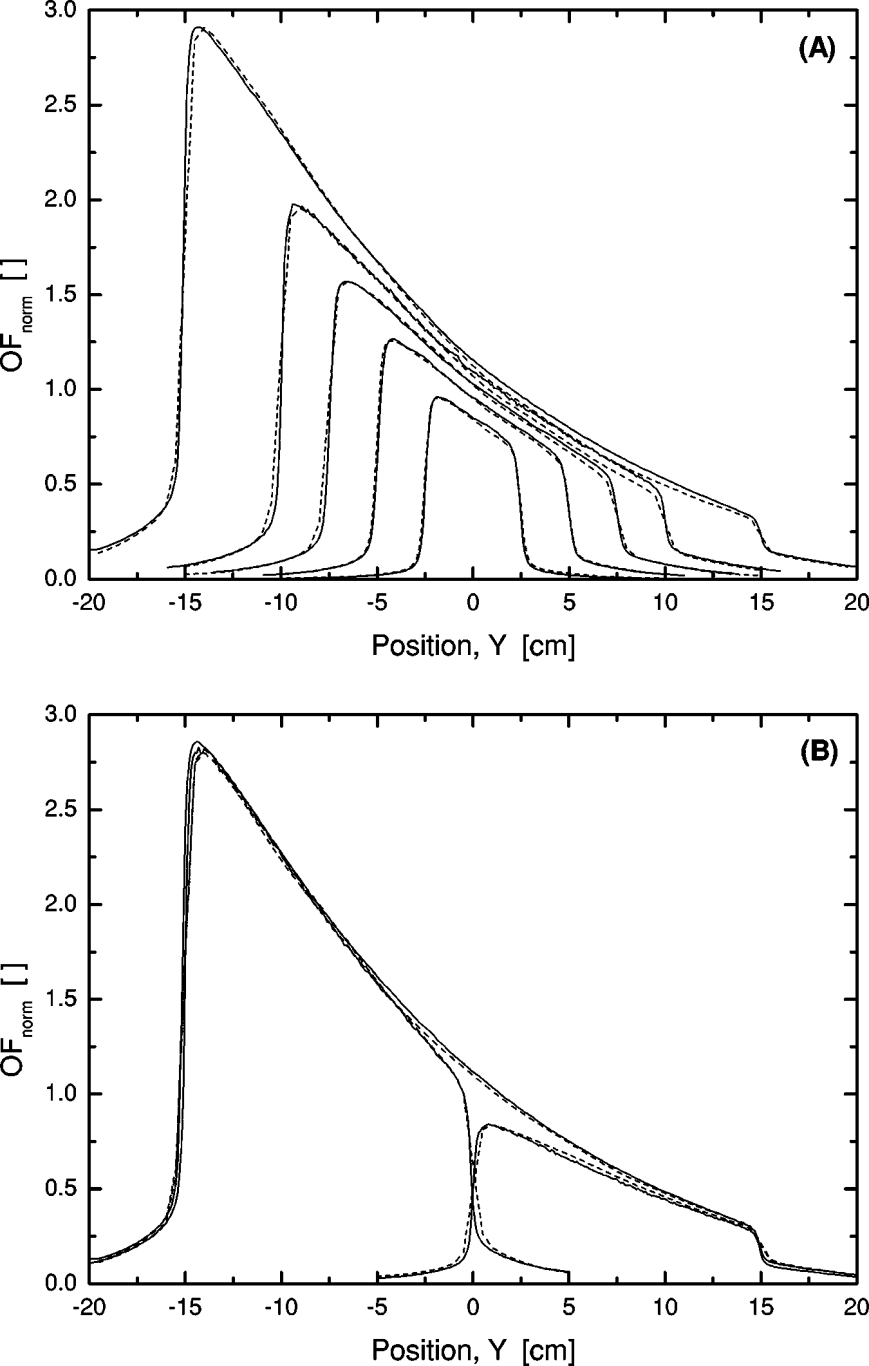
Profiles for 60° wedge fields at 10 cm depth, 6 MV, SSD=90 cm. (A) Square fields with field sides 30, 20, 15, 10, 5, and 3 cm (top to bottom). (B) Rectangular quadrant fields, 20×15 cm×cm with offset (15,–7.5) and (15,7.5) cm, as well as half field, 20×30 cm×cm with offset (15,0). The lines indicate measurements (solid —) and calculations (dashed ‐ ‐ ‐).

Figures 5(a) and 5(b) show comparisons for square wedge fields in the wedge direction, as well as a set of wedged quarter fields on each side of the major axis along the wedge direction. Compared to open beams, the results are less accurate when the large motorized wedge is introduced. The largest errors are associated with the largest field sizes where a discrepancy both in

output and profile shape can be seen. An underestimation of the modulation in the thicker parts of the wedge causes the profiles to have a slightly different shape as compared to the measurements.

The slope of the penumbra in the wedge direction, as can be seen in Figs. 5(a) and 5(b), differs slightly from measurements and is the result of a limitation in the TPS regarding voxel sizes. As dose calculation points, specified for line dose calculations, are deduced from linear interpolation

between density matrix voxels there may be a resolution problem when voxel sizes are relatively large and the dose gradient is high. This problem diminishes for the smallest field sizes where the voxel size in the *Y* direction is only 0.15 mm, as compared to 5.0 mm for the largest field.

### D. Off‐axis softening

**Figure 6:**
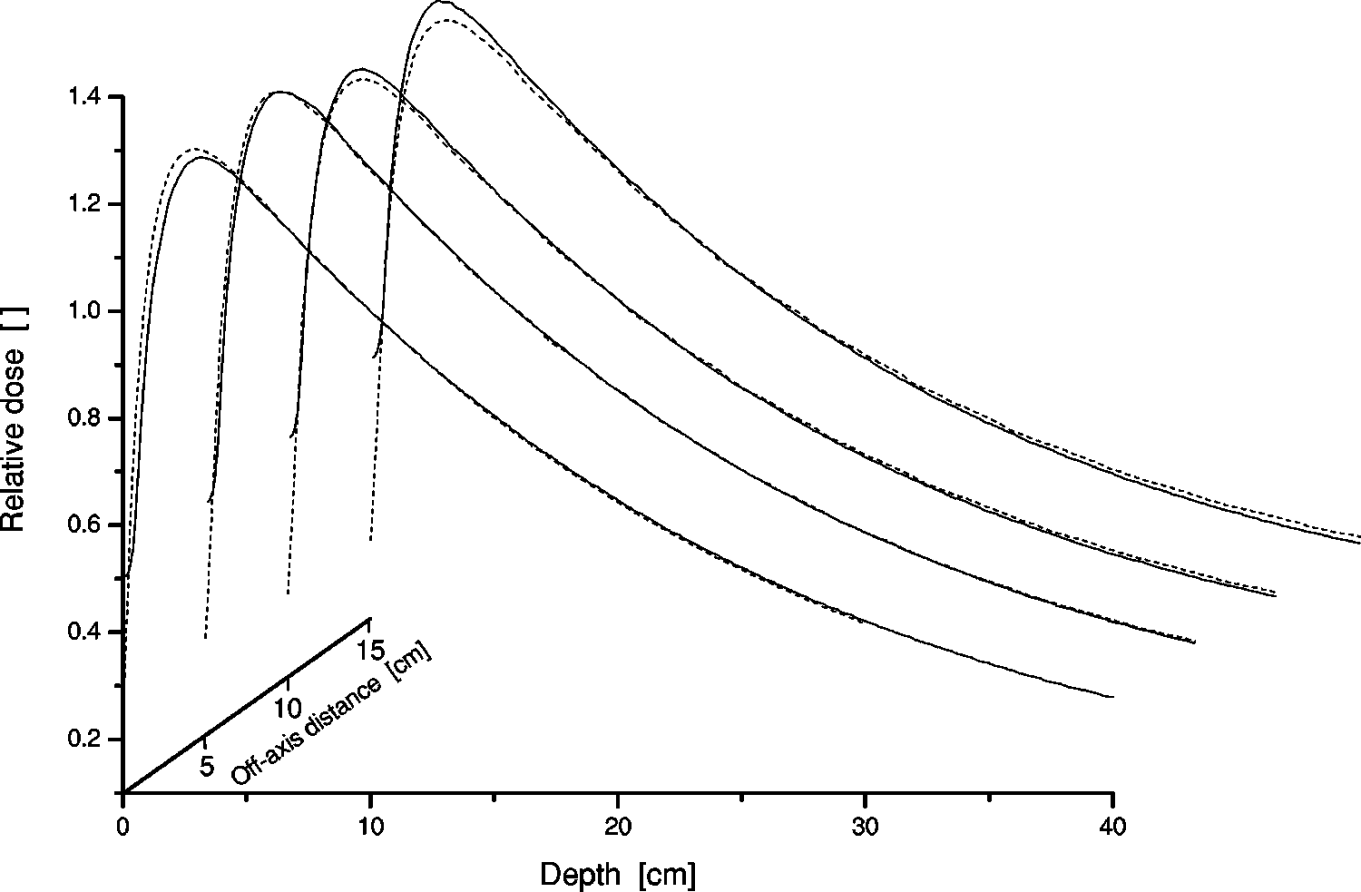
Divergent depth doses for 18 MV open 10×10 cm×cm field at SSD=90 cm with offset (0,0), (5,0), (10,0), and (15,0). Data has been normalized to 10‐cm depth along the fanline. The lines indicate measurements (solid —) and calculations (dashed ‐ ‐ ‐).

The effect of off‐axis softening has been investigated by positioning a 10 10 × 10 cm field with gradually increasing offset; 0, 5, 10, and 15 cm from the major axis. The largest effect of off‐axes softening is seen for higher energies and results plotted in Fig. 6 are thus for 18 MV. It can be seen that the slope of the measured fanline depth doses is steeper due to an energy decrease radially outwards where the beam traverses less flattening filter material. The deviation between calculated and measured depth doses is less than 2.5% at large depths for the curve furthest off‐axis.

### E. Partial phantom irradiation

**Figure 7:**
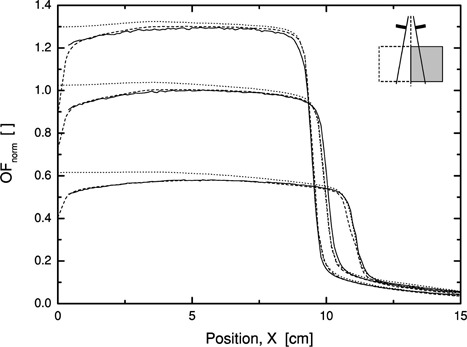
Profiles at 5, 10, and 20 cm for a 20×20 cm×cm field with half of the beam outside the phantom. 6 MV, SSD=90 cm. The lines indicate measurements (solid —) and calculations (dashed ‐ ‐ ‐). For comparison, the same field has been calculated under full scattering conditions (dotted ⋯).

Profiles at 5‐, 10‐, and 20‐cm depth have been obtained for an open 20 20 × 20 cm field with the central axis along the water surface with a gantry angle of 90°. Figure 7 shows the results for 6 MV with an SSD of 90 cm. Measurements within 3 mm from the surface have been neglected due to charged particle contamination from the phantom wall. Beyond this region, however, the agreement between measurements and calculations is excellent. For comparison, a profile calculated for a full scattering phantom has been included in the graph and it is seen that the phantom scattering effects are accounted for.

### F. Heterogeneities

**Figure 8:**
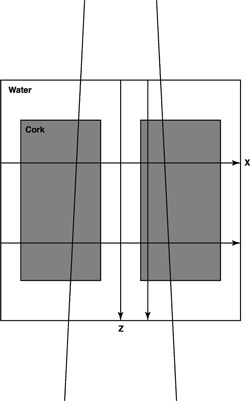
The geometry used for the Monte Carlo simulations. The white region has unit density and is set to be water $\left(\rho =1.0g/{\text{cm}}^{3}\right)$, whereas the gray area represents the low‐density region $\left(\rho =0.29g/{\text{cm}}^{3}\right)$ in cork. The lines with arrows indicate the position of the depth dose and profile calculations.

Due to experimental difficulties of measurements in heterogeneous media, the Monte Carlo method has been applied during this experiment. The mediastinum geometry, Fig. 8, of Knöös

**Figure 9:**
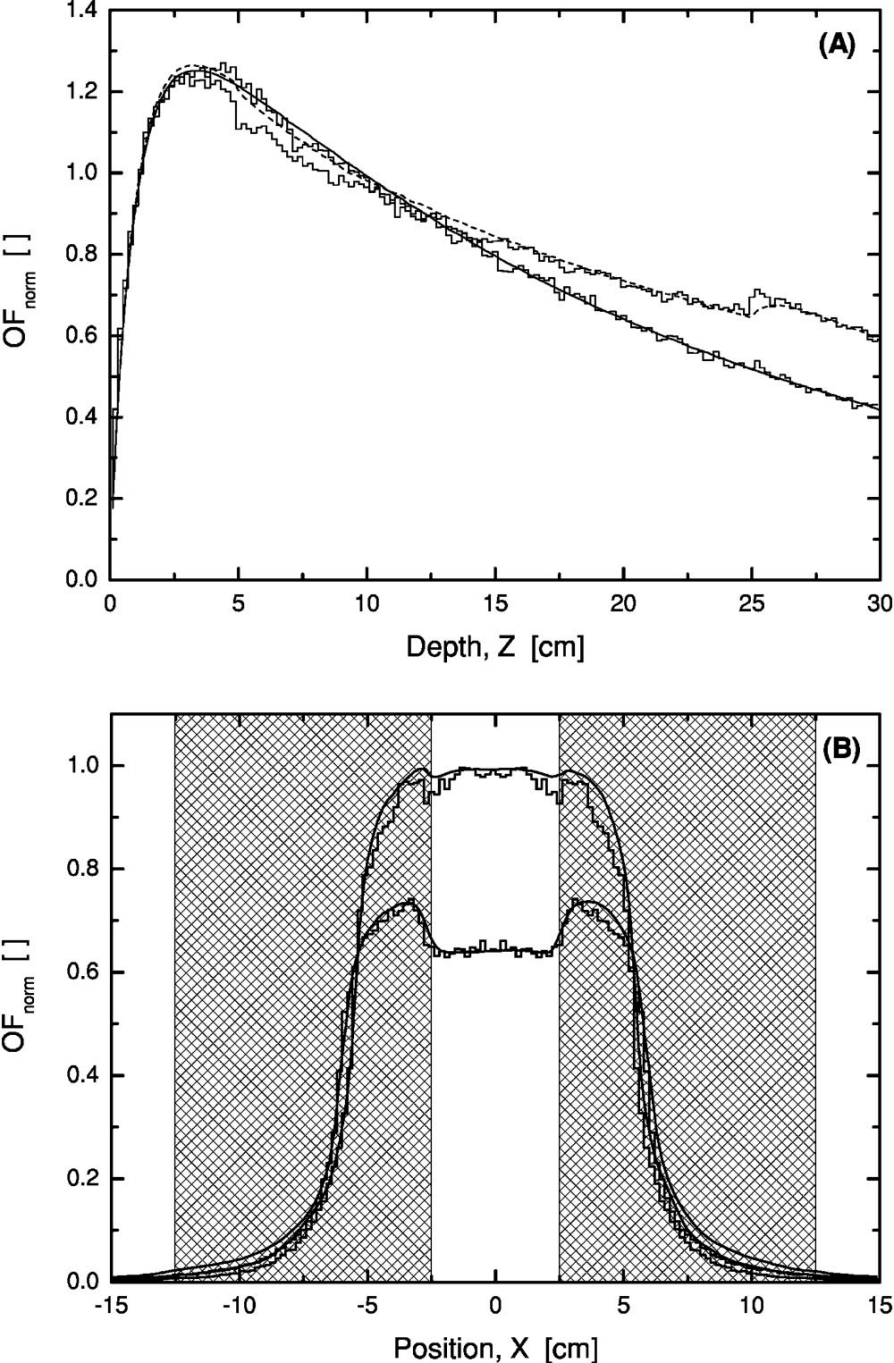
Depth doses and profiles for a mediastinum geometry (the hatched region indicate low‐density tissue) with an open 18 MV beam of field size 10 10 × 10 cm and SSD=100 cm. (A) Depth doses centrally through water and off‐axis (3.6,0) through low‐density tissue. (B) Profiles at 10‐ and 20‐cm depth. The histograms indicate Monte Carlo simulations and lines calculations (dashed ‐ ‐ ‐). The Monte Carlo simulations ignore charged particle contamination and the TPS calculated data have excluded this dose component.

*et al*.^7^ has been used for two open beams (6 and 18 MV) where the dose has been calculated using the EGS4 code, including the PRESTA algorithm. The simulations were made for monenergetic photons and assembled by means of superposition according to the spectrum of the beam quality. The number of histories varied between $16\times 10^{6}$ for 0.2 MeVto 3.$1\times 10^{6}$ for 18 MV. The standard deviation of the energy deposition was scored along the beam axis and dividing the number of histories into ten subgroups. The standard deviations obtained ranged from 2–6% were the higher number is associated with the lower energies. Figure 9(a) shows the output factor normalized results for 18 MV of depth doses centrally, as well as 3.6 cm off‐axis through the low‐density region. Profiles at 10 and 20 cm depth are shown in Fig. 9(b). A very good agreement between TPS calculated data and MC simulated data is seen. The rebuild up when exiting the low density region is calculated correctly, as well as the lateral dose spread close to the borders between the density regions. The discrepancies at shallower depths are due to the MC simulations lacking the electron contaminating dose component.

## IV. DISCUSSION

The pencil‐kernel model has previously shown to have some limitations in heterogeneous media, see Knöös *et al*.^7^ where it was pointed out that scatter from lateral heterogeneities, as well as disregarding the loss of electron equilibrium, may lead to significant errors. Other drawbacks are due to the spatially invariant pencil kernel leading to an underestimation of the output for larger fields, whereas the charged particle contamination model in some situations leads to unwanted overestimations of dose in the build up region implying problems for *in vivo* dosimetry.^5^

As expected, the major improvement of point kernel methods versus pencil kernel methods is for dose calculations in heterogeneous media. The scaling of the point kernel with the average electron density between the energy release point and dose deposition point allows a more accurate prediction of dose for a number of clinical situations (the lung region being the most prominent one) where pencil kernels yield large deviations for high‐energy photon beams.^7^ The recalculation of data for the geometry used by Knöös *et al*.^7^ showed [Fig. 9(a) and 9(b)] that the point kernel method is superior to pencil kernel methods especially for higher energies, when it comes to predicting the dose in the low‐density region. The region between the different densities caused by the large range of the high‐energy electrons is accurately modeled by an increase of the “penumbra” laterally between the two materials, as well as in the rebuild up for the water region in the depth dose through the low‐density region.

Other situations where the point kernel method is increasing the calculation accuracy over pencil kernels include off‐axis softening and patient/phantom related irradiated volume. Off‐axis softening has been implemented using a laterally varying attenuation coefficient for the primary dose component and the scatter part having a correlated radial dependence to the primary dose.^14^ ^,^ ^15^ This yields accurate dose calculations where the radial decrease in energy is manifested by depth dose curves with a decreasing slope when moving off axis, Fig. 6. It is only for the field positioned most outward where there is a tendency towards a small overestimation of the off‐axis effect. In the present implementation, no measurements are required for the off‐axis softening parameters. Instead the off‐axis softening is calculated based on generic data. The work presented by Tailor *et al*.^20^ is used and this is a fit to a number of accelerators where it should be possible to improve dose calculations off‐axis by applying machine specific measurements.

In clinical situations the contour of the patient is changing over the field aperture. The pencil kernels have properties that neglect the full influence of the patient scattering volume, as has been shown by, e.g., Hurkmans *et al*.^6^ Situations where this is prominent include the head and neck region and breast treatments with opposing tangential fields. The point kernel algorithm taking into account the full extension of the patient or phantom contour (Fig. 7) avoids this problem. This also applies to the exit side of a beam where the loss of backscattering material is accounted for. An improvement in the calculation accuracy of exit dose data for *in vivo* dosimetry purposes based on TLDs or diodes should thus be possible to achieve.

Results presented here show good agreement with measurements in homogeneous as well as heterogeneous media, and most cases are within established limits.^18^ ^,^ ^21^ ^,^ ^22^ Ahnesjö and Aspradakis^18^ give an accuracy level of 3% in the TPS dose calculation in order to achieve an overall uncertainty of 5% in the absorbed dose to the patient. There exist, however, situations where the calculations are outside these limits and this includes wedge output and dose calculations in the build up region. Wedges are implemented in the TPS by adapting a modulation matrix to a measured lateral dose distribution as mentioned in Table I and applying quality dependent correction factors for the beam hardening effects on primary and scatter components introduced by the wedge.^23^ In most cases the results are satisfactory, but situations exist where larger discrepancies are present. This has previously been reported^24^ and it was found that for wedges positioned high up in the treatment head, such as motorized wedges, the uncertainty in dose calculation accuracy is larger than for wedges located further down in the treatment head (manually inserted mechanical wedges). This may also be attributed to the fact that the motorized wedges usually are physically much thicker at the central axis as they are designed to allow for 30‐cm fields in the wedge direction and the amount of scatter generated in the wedge may be underestimated. It should also be noted that the errors in clinical situations are smaller as the motorized wedge is mostly used during fractions of the overall beam‐on time.

The dose in the build up region is evaluated by calculating the difference between point kernel calculated depth dose data and the measurements for this region. The difference is attributed to charged particle contamination released by the flattening filter, collimators, and other beam modifiers and is modeled by an exponential with three parameters fitted to the difference of the measured and calculated standard set of depth doses. Open as well as modulated beams by wedges or compensators all have individual parameter sets.

Although inadequate measurement techniques may influence the results, it is clear that the implemented model gives unsatisfactory results, especially outside the characterization domain. However, as more information regarding the physics of the build up region is being presented, it should be possible to find better algorithms that more accurately would predict the dose in this region. Several authors have already presented data that may improve dose calculation results significantly.^25^ ^,^ ^26^

## V. CONCLUSION

A point kernel model, collapsed cone, has been incorporated in a commercial treatment planning system and an investigation where measured and TPS calculated data are compared in homogeneous media has been conducted. For heterogeneous media, a comparison has been made with MC generated data. The collapsed cone calculations were found feasible for clinical use as calculations agreed to experimental data within 3% for most of the tested geometries. Dose calculation problems associated with the invariant pencil kernel algorithm (lack of off‐axis softening, geometry dependence, heterogeneities) have been considerably improved in the implemented point kernel and should be beneficial in a number of clinical situations. However, some geometries are still present where the accuracy may be considered inadequate. These are not specific to the collapsed cone implementation as these situations include large wedge fields and dose in the build up region.

## ACKNOWLEDGMENTS

Anders Ahnesjö and Mikael Saxner, MDS Nordion Therapy Systems, are greatly acknowledged for valuable comments. Anders Ahnesjö is further acknowledged for supplying the Monte Carlo simulations.

## DOSE CALCULATION TIME

All three‐dimensional (3D) dose calculations in the TPS, regardless of particle type, are conducted on an internal calculation matrix, the density matrix. This matrix represents the tissue properties of the patient (or phantom). For tissue information with computerized tomography (CT) as a base, the Hounsfield units are compressed into the range [‐128,127] according to

$$HU_{\text{TMS}}=\left(\frac{HU+1000}{16}\right)-128,$$

where *HU* is the Hounsfield unit as acquired on the CT scanner and HUTMS is the corresponding compressed value representing the tissue in the TPS.

Not all of the available 256 values are used for tissue representation. At the high end, 16 values are reserved for special purpose materials. These may be used for foreign materials in the human body, e.g., prostheses manufactured in steel‐ or titanium‐related materials, while others are used for constructing phantoms in the TPS, e.g., PMMA, polystyrene, and cork. While CT based data is assigned a tissue property depending on the Hounsfield value when generating the density matrix, the phantom materials are assigned values based on the composition assigned by the user.

The density matrix is used for the dose calculations as the representation of the patient. For each of the voxels, dose will be calculated at its midpoint. The user defined resolution for dose calculations, the values according to the dose grid, are calculated by a 3D interpolation in the density matrix. From a calculation time aspect this is a minor task as most of the time is spent on ray tracing within the density matrix. The calculation time, *t*, is proportional to

$$t\propto N_{X}N_{Y}N_{Z}N_{\text{cones}},$$

where *N* ^*X*^, *N* ^*Y*^, *N* ^*Z*^ is the number of voxels in the *X, Y*, and *Z* direction, respectively, and $N_{\text{cones}}$ is the number of directions used in the discretization of the kernel.

## References

[1] C‐S. Chui , ‘A method for three‐dimensional gamma ray dose calculations in heterogeneous media and its application in radiation therapy,” Ph.D thesis, Columbia University, 1985.

[2] A. Ahnesjö , ‘Dose calculation methods in photon beam therapy using energy deposition kernels,” Ph.D. thesis, Stockholm University, 1991.

[3] H. H. Liu , ‘Anew convolution/superposition dose calculation method for external photon beam radiation therapy using beam modifiers and/or independent collimators,” Ph.D thesis, Mayo Graduate School, Rochester, 1997.

[4] P. Storchi , ‘Calculation methods for the prediction of absorbed dose in radiotherapy physics,” Ph.D thesis, Technical University, Delft, 1999.

[5] T. Knöös , C. Ceberg , L. Weber , and P. Nilsson , “The dosimetric verification of a pencil beam based treatment planning system,” Phys. Med. Biol. 39, 1609–1628 (1994). 15551534 10.1088/0031-9155/39/10/007

[6] C. Hurkmans , T. Knöös , P. Nilsson , G. Svahn‐Tapper , and H. Danielsson , “Limitations of a pencil beam approach to photon dose calculations in the head and neck region,” Radiother. Oncol. 37, 74–80 (1995). 8539461 10.1016/0167-8140(95)01609-k

[7] T. Knöös , A. Ahnesjö , P. Nilsson , and L. Weber , “Limitations of a pencil beam approach to photon dose calculations in lung tissue,” Phys. Med. Biol. 40, 1411–1420 (1995). 8532755 10.1088/0031-9155/40/9/002

[8] C. Hurkmans , T. Knöös , and P. Nilsson , “Dosimetric verification of open asymmetric photon fields calculated with a treatment planning system based on dose‐to‐energy‐fluence concepts,” Phys. Med. Biol. 41, 1277–1290 (1996). 8858720 10.1088/0031-9155/41/8/003

[9] L. Weber , A. Ahnesjö , P. Nilsson , M. Saxner , and T. Knöös , “Verification and implementation of dynamic wedge calculations in a treatment planning system based on a dose‐to‐energy‐fluence formalism,” Med. Phys. 23, 307–316 (1996). 8815372 10.1118/1.597797

[10] A. A. van't Veld , “Analysis of accuracy in dose and position in calculations of a treatment planning system for blocked photon fields,” Radiother. Oncol. 45, 245–251 (1997). 9426118 10.1016/s0167-8140(97)00133-3

[11] H. Hansson , P. Björk , T. Knöös , and P. Nilsson , “Verification of a pencil beam based treatment planning system: output factors for open photon beams shaped with MLC or blocks,” Phys. Med. Biol. 44, N201–N207 (1999). 10495127 10.1088/0031-9155/44/9/402

[12] A. Ahnesjö , M. Saxner , and A. Trepp , “A pencil beam model for photon dose calculation,” Med. Phys. 19, 263–273 (1992). 1584117 10.1118/1.596856

[13] A. Ahnesjö , “Collapsed cone convolution of radiant energy for photon dose calculation in heterogeneous media,” Med. Phys. 16, 577–592 (1989). 2770632 10.1118/1.596360

[14] M. Saxner and A. Ahnesjö , “Implementation of the collapsed cone method for clinical beam qualities,” Med. Phys. 25, A185 (1998).

[15] A. Ahnesjö , M. Saxner , and I. Thorslund , “Modelling of photon beam spectral variations,” in Proceedings of the Int. Conf. on the Use of Computers in Radiation Therapy, XIII ICCR (Heidelberg, Germany, 2000), edited by Schlegel W. and Bortfeld T., Springer, Berlin, Germany, 2000, pp. 227–228.

[16] A Ahnesjö , “Cone discretization for the collapsed cone algorithm” in Proceedings of the Int. Conf. on the Use of Computers in Radiation Therapy, XII ICCR, Salt Lake City, USA, 1997, edited by Leavitt D. D. and Starkschall G. Medical Phyics Publishing, Madison, WI, 1997, pp. 114–116.

[17] L. Weber , P. Nilsson , and A. Ahnesjö , “Build‐up cap materials for measurement of photon head‐scatter factors,” Phys. Med. Biol. 42, 1875–1886 (1997). 9364584 10.1088/0031-9155/42/10/002

[18] A. Ahnesjö and M. M. Aspradakis , “Dose calculations for external photon beams in radiotherapy,” Phys. Med. Biol. 44, R99–R155 (1999). 10588277 10.1088/0031-9155/44/11/201

[19] International Electrotechnical Commision , ‘Medical electrical equipment. Medical electron accelerators in the range 1 MeV to 50 MeV ‐ Guidelines for functional performance characteristcs,” IEC Technical Report 977,” (IEC, Geneva, 1989).

[20] R. C. Tailor , V. M. Tello , C. B. Schroy , M. Vossler , and W. F. Hanson , “A generic off‐axis energy correction for linac beam dosimetry,” Med. Phys. 25, 662–667 (1998). 9608476 10.1118/1.598249

[21] J. Van Dyk , R. B. Barnett , J. E. Cygler , and P. C. Shragge , “Commissioning and quality assurance of treatment planning computers,” Int. J. Radiat. Oncol., Biol., Phys. 26, 261–273 (1993). 8491684 10.1016/0360-3016(93)90206-b

[22] B. Fraass , K. Doppke , M. Hunt , G. Kutcher , G. Starkschall , R. Stern , and J. Van Dyk , American Association of Physicists in Medicine, Radiation Therapy Task Group 53 : “Quality assurance for clinical radiotherapy treatment planning,” Med. Phys. 25, 1773–1829 (1998). 9800687 10.1118/1.598373

[23] A. Ahnesjö , L. Weber , and P. Nilsson , “Modelling transmission and scatter for photon beam attenuators,” Med. Phys. 22, 1711–1720 (1995). 8587523 10.1118/1.597534

[24] L. Weber , A. Ahnesjö , and I. Kivultjik , “Clinical accuracy of a pencil kernel model for wedge dose calculations,” Radiother. Oncol. 48, S134 (1998).

[25] R. Sjögren and M. Karlsson , “Electron contamination in clinical high energy photon beams,” Med. Phys. 23, 1873–1881 (1996). 8947901 10.1118/1.597750

[26] A. R. Hounsell and J. M. Wilkinson , “Electron contamination and build‐up doses in conformal radiotherapy fields,” Phys. Med. Biol. 44, 43–55 (1999). 10071874 10.1088/0031-9155/44/1/005

